# Clinical Lectures on Diseases of the Eye

**Published:** 1865-04

**Authors:** E. L. Holmes

**Affiliations:** Chicago, Lecturer on Diseases of the Eye and Ear in Rush Medical College, and Surgeon of the Chicago Charitable Eye and Ear Infirmary


### CLINICAL LECTURES ON DISEASES OF THE EYE.

By E. L. HOLMES, M. D., of Chicago,
*Lecturer on Diseases of the Eye and Ear in Rush Medical College, and Surgeon of the Chicago Charitable Eye and Ear Infirmary.*

### CHOROIDITIS.

*Gentlemen :*—The diseases of the Choroid, as now understood from investigations with the ophthalmoscope and microscope, may be divided into two classes: those in which an undue hardness of the globe, caused by an abnormal increase of its fluids, is a characteristic symptom ; and those in which this hardness is not a characteristic symptom.

Cases of the first class, to which the term "glaucomatous" is often given, are peculiar to patients somewhat advanced in years. Although glaucoma is apparently a very rare disease at the west, its discussion involves a few very important principles, and for this reason I shall make it the special subject of the next lecture.

Diseases of the Choroid, belonging to the second class, are not unfrequent, and embrace many cases which were included, previous to the discovery of the ophthalmoscope, in the indefinite terms, amaurosis and amblyopia.

One of the most common forms of Choroiditis is intimately related with disease of the retina, and is known as retinitis pigmentosa, although Choroiditis pigmentosa would seem to be the most appropriate term. The only symptom of this disease experienced by the patient is usually, a more or less gradual loss of vision. There is generally neither pain nor any abnormal appearance, as seen by simple inspection. Occasionally the pupil will be found either oval in form or sluggish under the influence of alternate light and shade. Belladonna seems to dilate the pupil more slowly than in health. The disease is peculiar to youth and early manhood, and generally, it is stated, affects both eyes.

Upon examination with the ophthalmoscope, the lens, vitreous humor, and cornea are almost always found transparent. The choroid, especially its posterior portions, present peculiar changes, which are well represented in the plates of Jæger and Liebrich.

In some cases the pigment layer is, to a greater or less extent, absorbed, exposing to view, in irregular patches, the larger vessels of the choroid, which appear as quite broad, yellow lines, beautifully curved and looped together. Instead of being thus absorbed, the pigment will often be found deposited in increased quantities in irregular, jet black patches. With these appearances there are, not unfrequently, more or less extensive, thin yellow exudations. The portions of the choroid, thus affected with yellow deposit, are very often surrounded by narrow black lines of pigment. These three abnormal changes may take place in the same eye, varying in different cases, as regards their relative extent. The different shades of color and combination of forms, as seen with the ophthalmoscope, are often very beautiful.

The disease is generally, either as cause or complication, accompanied with an abnormal condition of the retina and optic nerve, as shown by changes in the retinal vessels and the papilla.

Scarcely anything is known regarding the cause of this affection. Although a few cases may be referred to a scrofulous or syphilitic condition, it does not in the majority of cases seem to depend upon any particular diathesis. A few cases have been observed in children of consanguineous parents. Too few observations have been made, however, to prove the relation of cause and effect in these cases.

The progress of the disease is usually very slow, and the prognosis as regards improvement of vision grave. Nothing of much practical value is known regarding the treatment of this disease. Everything possible should be done on the part of the patient to preserve good health and in no way to fatigue the eyes.

Another form of choroiditis generally observed in children is characterized by the presence of an organized tissue, extending from the choroid, in the form of a tumor, which causes absorption of the vitreous humor, and finally fills the whole globe. This has been called hyperplastic inflammation of the choroid.

The symptoms, as regards pain and congestion, are very slight in some cases, and in others very marked. Vision even in the early stages of the disease, is destroyed. As the growth increases in size, it can be seen with the unassisted eye, as a glistening, yellowish white tumor behind the iris, which somewhat resembles the encephaloid tumor of the retina peculiar to children. It is not, however, malignant, and is not prone to return in the adjacent tissues when the globe has been extirpated. The diagnosis between this form of non-malignant growth and true cancerous disease is sometimes very difficult.

The tumor is composed of long spindle shaped cells, with an intercellular substance, containing blood vessels.

As the disease progresses it produces absorption of the intraocular tissues and finally terminates in atrophy, which may occur either with or without suppuration. In the majority of cases suppuration causes sloughing of the cornea and destruction of the tumor itself. Occasionally suppuration fails to produce this result, the size of the tumor and the amount of purulent discharge both increasing until the patient finally succumbs.

In cases of atrophy without pain, and sympathetic inflammation of the other eye, there is little call for treatment. But in the cases just mentioned, with exhaustive suppuration, the removal of the eye seems to be the only alternative.

Choroiditis of a more or less active form, and often tending to the formation of pus, is not unfrequently the result of several abnormal conditions, not only of the eye but of the system. The iris is almost always implicated in this form of the disease. There is pain in and around the eye varying very much, however, in intensity in different cases; congestion of the subconjunctival and conjunctival vessels; discoloration of the iris with sluggishness and irregularity of the pupil, and very speedy loss of sight, with opacity of the internal refracting media of the eye. There is sometimes an increased hardness of the globe, although this is not a characteristic symptom of the disease.

The inflammation may arise from injuries, as from pieces of steel or percussion cap penetrating the globe, severe blows, wounds of the cornea and sclerotic. It may follow operations for cataract, either by extraction or depression. Large penetrating ulcers of the cornea, by reducing the normal intraocular pressure, may occasionally cause passive congestion of the choroid, terminating in inflammation.

The iris and choroid of one eye may become affected by sympathy when the other eye is suffering from choroiditis, especially when produced by foreign substances within the globe by a dislocated lens, or by severe injury of the ciliary bodies. Choroiditis is occasionally a complication with suppurating or gangrenous wounds of the body, with puerperal fever, typhoid fever, variola, measles. Neglected iritis, if subject to relapses, and attended with adhesions of the iris to the lens, is very liable to be accompanied with choroiditis.

The prognosis in nearly all these cases, especially if there is tendency to the formation of pus, is unfavorable. The most careful attention to diet, rest, the use of alteratives and absorbents, seem scarcely to be of any avail.

The large majority of cases require either the removal of a piece of the iris, or the section of the ciliary muscle, as proposed by Hancock. If the disease is caused by the pressure of a foreign substance in the globe, or by a dislocated lens, efforts should be made to remove them, provided they can be seen. For the purpose of saving the other eye, it is not unfrequently necessary to resort to the extirpation of the globe originally diseased. This operation is perhaps demanded whenever there are symptoms of sympathetic inflammation, as photophobia, with loss of sight, and congestion of the deeper vessels of the conjunctiva.

